# Molecular and Thermodynamic Mechanisms of the Chloride-dependent Human Angiotensin-I-converting Enzyme (ACE)*

**DOI:** 10.1074/jbc.M113.512335

**Published:** 2013-12-02

**Authors:** Christopher J. Yates, Geoffrey Masuyer, Sylva L. U. Schwager, Mohd Akif, Edward D. Sturrock, K. Ravi Acharya

**Affiliations:** From the ‡ Institute of Infectious Disease and Molecular Medicine and Division of Medical Biochemistry, University of Cape Town, Observatory 7935, South Africa and; the § Department of Biology and Biochemistry, University of Bath, Claverton Down, Bath BA2 7AY, United Kingdom

**Keywords:** Biophysics, Crystallography, Hypertension, Protein Structure, Thermodynamics, Angiotensin-1-converting Enzyme, Chloride Ion Activation, Enzymology

## Abstract

Somatic angiotensin-converting enzyme (sACE), a key regulator of blood pressure and electrolyte fluid homeostasis, cleaves the vasoactive angiotensin-I, bradykinin, and a number of other physiologically relevant peptides. sACE consists of two homologous and catalytically active N- and C-domains, which display marked differences in substrate specificities and chloride activation. A series of single substitution mutants were generated and evaluated under varying chloride concentrations using isothermal titration calorimetry. The x-ray crystal structures of the mutants provided details on the chloride-dependent interactions with ACE. Chloride binding in the chloride 1 pocket of C-domain ACE was found to affect positioning of residues from the active site. Analysis of the chloride 2 pocket R522Q and R522K mutations revealed the key interactions with the catalytic site that are stabilized via chloride coordination of Arg^522^. Substrate interactions in the S2 subsite were shown to affect chloride affinity in the chloride 2 pocket. The Glu^403^-Lys^118^ salt bridge in C-domain ACE was shown to stabilize the hinge-bending region and reduce chloride affinity by constraining the chloride 2 pocket. This work demonstrated that substrate composition to the C-terminal side of the scissile bond as well as interactions of larger substrates in the S2 subsite moderate chloride affinity in the chloride 2 pocket of the ACE C-domain, providing a rationale for the substrate-selective nature of chloride dependence in ACE and how this varies between the N- and C-domains.

## Teaser

**Background:** The angiotensin-1-converting enzyme (ACE) is a zinc protease unique in its chloride ion dependence.

**Results:** Key substrate interactions with the catalytic site affect the structure and thermodynamics of ACE, which modulate chloride ion-dependent hydrolysis.

**Conclusion:** Substrate composition influences chloride affinity in ACE.

**Significance:** The interdependence of ACE substrate composition, structure, and thermodynamics influence its chloride ion activation in biology.

## Introduction

Angiotensin I-converting enzyme (ACE^5^; EC 3.4.15.1) is a zinc metallopeptidase that functions as a dipeptidyl carboxypeptidase, catalyzing the hydrolysis of a wide range of functional peptides. Its main role is within the renin-angiotensin system, which regulates blood pressure and renal homeostasis and thus is an important drug target for the treatment of hypertension and other cardiovascular and renal diseases (1–3). The systemic renin-angiotensin system relies on a series of proteolytic events involving circulating renin/prorenin and somatic ACE (sACE), present on the external surface of endothelial cells, to process angiotensinogen into angiotensin II (AngII), a hypertensive and mitogenic octapeptide. sACE catalyzes the last step in the production of AngII by cleaving the C-terminal dipeptide of AngI (4). The main form of mammalian sACE consists of two very similar domains (N- and C-domains) each possessing a functional catalytic site (1, 2). There is also a smaller testicular form of ACE (tACE), which is transcribed from the same gene and identical to the C-domain of ACE. The mammalian ACE gene arose from a gene duplication event during the course of vertebrate evolution (5).

The active site for both domains is contained within the large central groove, with the signature HE*XX*H zinc-binding motif residing on the α15 helix (6, 7). Access to this active site is severely limited, with a small pore in the N-terminal chamber or an occluded slot in the C-terminal chamber providing the only theoretical entry and suggests that a degree of flexibility in the domain movements is required for substrate access (7, 8). The catalytic mechanism has been suggested based on comparison with work done on the structurally analogous zinc metalloprotease, thermolysin, and is proposed to occur via a general base mechanism, whereby a nucleophilic water molecule or hydroxide ion attacks the carbonyl carbon of the scissile bond (9, 10).

Two chloride ions are found buried in the structure of tACE and separated by a distance of 20.3 Å (6). The first chloride (Cl1), 20.7 Å away from the zinc, is bound by four ligands, namely Arg^186^, Trp^485^, Arg^489^, and water, and is encapsulated by a hydrophobic shell of four tryptophans (6). The N-domain crystal structures show no chloride bound in this pocket, with the only difference in residue composition in the pocket being His^164^, which corresponds to Arg^186^ in tACE (7). The second chloride ion (Cl2), which is observed in both the C- and N-domain structures, is located much closer to the active site zinc at 10.4 Å and is coordinated by Arg^522^, Tyr^224^ (Arg^500^ and Tyr^202^ in the N-domain), and a water molecule (6).

ACE activity is dependent on chloride ions, with the degree of dependence being substrate-specific (11). ACE is situated mainly on the surface of endothelial tissue, an environment where blood plasma chloride levels rarely fluctuate from the 100 mm average, sufficient for ACE to be considered fully active in angiotensin I hydrolysis (11). However, ACE has been found in other non-vascular tissues, including kidney, brain, bone marrow, pancreas, and adipose tissues (12), where the chloride levels can vary substantially, and also intracellularly (13), where the chloride concentration is ∼5 mm (14). These varying concentrations would have an impact on ACE catalytic efficiency.

Variance in chloride activity is also seen in inhibitor binding. The difference in affinities for the ACE inhibitors trandolaprilat, enalaprilat, and lisinopril between the N- and C-domains is greater at high chloride concentration (300 mm), whereas for captopril, the difference is larger at low chloride concentration (20 mm) (15). The variability in chloride-mediated enhancement of inhibitor affinity may have potential applications in design of inhibitors targeting tissue-specific ACE activity. Indeed, a full delineation of the exact structural and mechanistic aspects of ACE chloride dependence for each domain could be important for the development of domain-selective inhibitors.

In this study, key single point mutations were generated to investigate the mechanism of chloride activation and substrate specificity. Activity of these mutants was characterized using an isothermal titration calorimetry (ITC)-based assay providing in depth information on the enzyme thermodynamic and kinetic parameters under varying chloride concentrations. Furthermore, the high resolution x-ray crystal structures of these mutants were determined, revealing the key molecular interactions involved in chloride binding. Together, these data identified the close relationship between the nature of the substrate and chloride activation through the chloride 2 pocket and the S2 subsite. The role for the chloride 1 pocket remains elusive and could be mainly structural. A key salt bridge specific to C-domain ACE between Glu^403^ and Lys^118^ was shown to stabilize the hinge-bending region and reduce chloride affinity by constraining the chloride 2 pocket. This work provides a rationale for the substrate-selective nature of chloride dependence in ACE and how this varies between the N- and C-domains.

## EXPERIMENTAL PROCEDURES

### 

#### 

##### Chemicals

All chemicals were purchased from Merck and Sigma-Aldrich. Benzyloxycarbonyl-l-Phe-l-His-Leu (Z-FHL) was purchased from Bachem AG (Bubendorf, Switzerland).

##### Mutations

**FIGURE 1.**
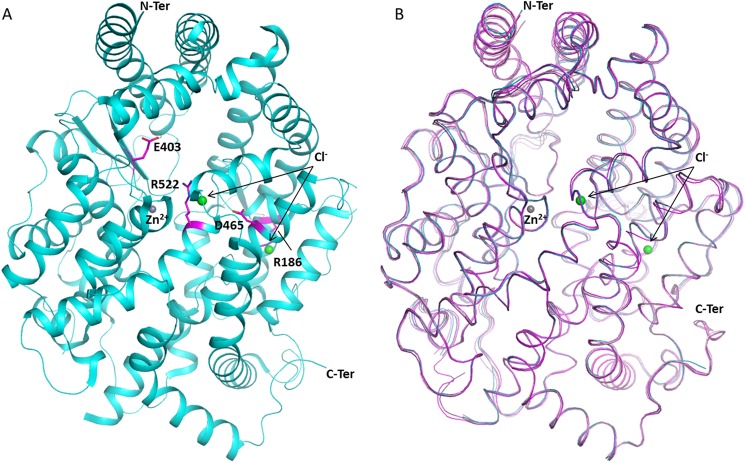
**Overall structure of C-domain ACE mutants.** *A*, location of mutated residues Glu^403^, Asp^465^, and Arg^522^ (*magenta*) in C-domain ACE (*cyan*; Protein Data Bank code 1O8A (6)), with chloride and zinc ions shown as *spheres* (*green* and *gray*, respectively). *B*, superposition of C-domain ACE (*cyan*; Protein Data Bank code 1O8A (6)) with the mutant structures E403R (*light pink*), D465T (*pink*), R522K (*violet*), and R522Q (*purple*).

A fully soluble, minimally *N*-glycosylated version of human tACE, tACEΔ36-g13sol (16) (referred to as the C-domain hereafter), truncated after Ser^625^ and lacking the 36 *O*-glycosylated N-terminal residues (17), was cloned into the BamHI and NotI restriction sites of pcDNA3.1(+) (Invitrogen) in order to facilitate expression in mammalian cells. A series of C-domain mutants were generated (Table 1 and Fig. 1) using a cassette-based subcloning strategy, as described previously (16), whereby PCR-based site-directed mutagenesis was used to introduce single amino acid mutations into fragments of the C-domain, contained within pGEM11Zf(+) (Promega), which were sequenced bidirectionally to verify the mutation and then subcloned into the C-domain. Human soluble N-domain Asp^629^ (18), containing residues 1–629, was previously cloned into EcoRI and XbaI restriction sites of pcDNA 3.1(+)(Invitrogen) for expression.

**TABLE 1:** **Site-directed mutagenesis of C-domain ACE** Shown is the location of the mutations with respect to potential role, the name of the mutation, and residue in the C-domain that was mutated along with its numerical position, identity of the amino acid if it is being converted to its corresponding N-domain residue, and the identity of the residue if that mutation is completely novel (*i.e.* same in both domains).

Location	Mutant name	C-domain	N-domain	Non-domain mutation
Chloride 1 pocket	R186H	Arg^186^	His	
S2 pocket (chloride channel)	E403R	Glu^403^	Arg	
Chloride 2 pocket	D465T	Asp^465^		Thr
	R522K	Arg^522^		Lys
	R522Q	Arg^522^		Gln

##### Enzymes

N- and C-domain human ACE proteins were generated by expression in cultured mammalian CHO cells (human ACE) and purified to homogeneity as described previously (16, 19).

##### X-ray Crystallography

Crystals were obtained with 1 μl of the C-domain mutant sample (5–10 mg/ml in 50 mm HEPES, pH 7.5, 0.1 mm PMSF) mixed with an equal volume of reservoir solution (100 mm MIB buffer, pH 4.0, 10 μm zinc sulfate, 5% glycerol, and 15% PEG 3350) and suspended above the well as a hanging drop. Diffraction quality crystals of C-domain mutants appeared after ∼3–5 days. The R522K·captopril complex was obtained by co-crystallization with a 1 mm concentration of the ligand.

X-ray diffraction data for C-domain mutants were collected on PX station IO2, IO4, and IO4-1 at the Diamond Light Source (Oxon, UK). 25% PEG 3350 was added to the drop as a cryoprotectant to keep the crystal at constant temperature (100 K) under the liquid nitrogen jet during data collection. For each mutant, 100–150 images were collected by using a PILATUS-2M, -6M (Dectris, Switzerland) or a Quantum-315 CCD (Area Detector Systems Corp., Poway, CA) detector. Raw data images were processed and scaled with XDS (20), MOSFLM (21), or XIA2 and SCALA using the CCP4 suite (22). Initial phases for structure solution were obtained using the molecular replacement routines of the PHASER program (23). The atomic coordinates of native C-domain (Protein Data Bank code 1O8A (6)) were used as a search model for structure determination. The resultant models were refined using REFMAC5 (24). Five percent of reflections were separated as an *R*_free_ set and used for cross-validation (25). Manual adjustments of the model were carried out using COOT (26). Water molecules were added at positions where *F_o_* − *F_c_* electron density peaks exceeded 3σ, and potential hydrogen bonds could be made. Validation was conducted with the aid of the program MOLPROBITY (27). Crystallographic data statistics are summarized in Table 2. All figures were drawn with PyMOL (Schrödinger, LLC, New York) and rendered with POV-ray.

**TABLE 2:**
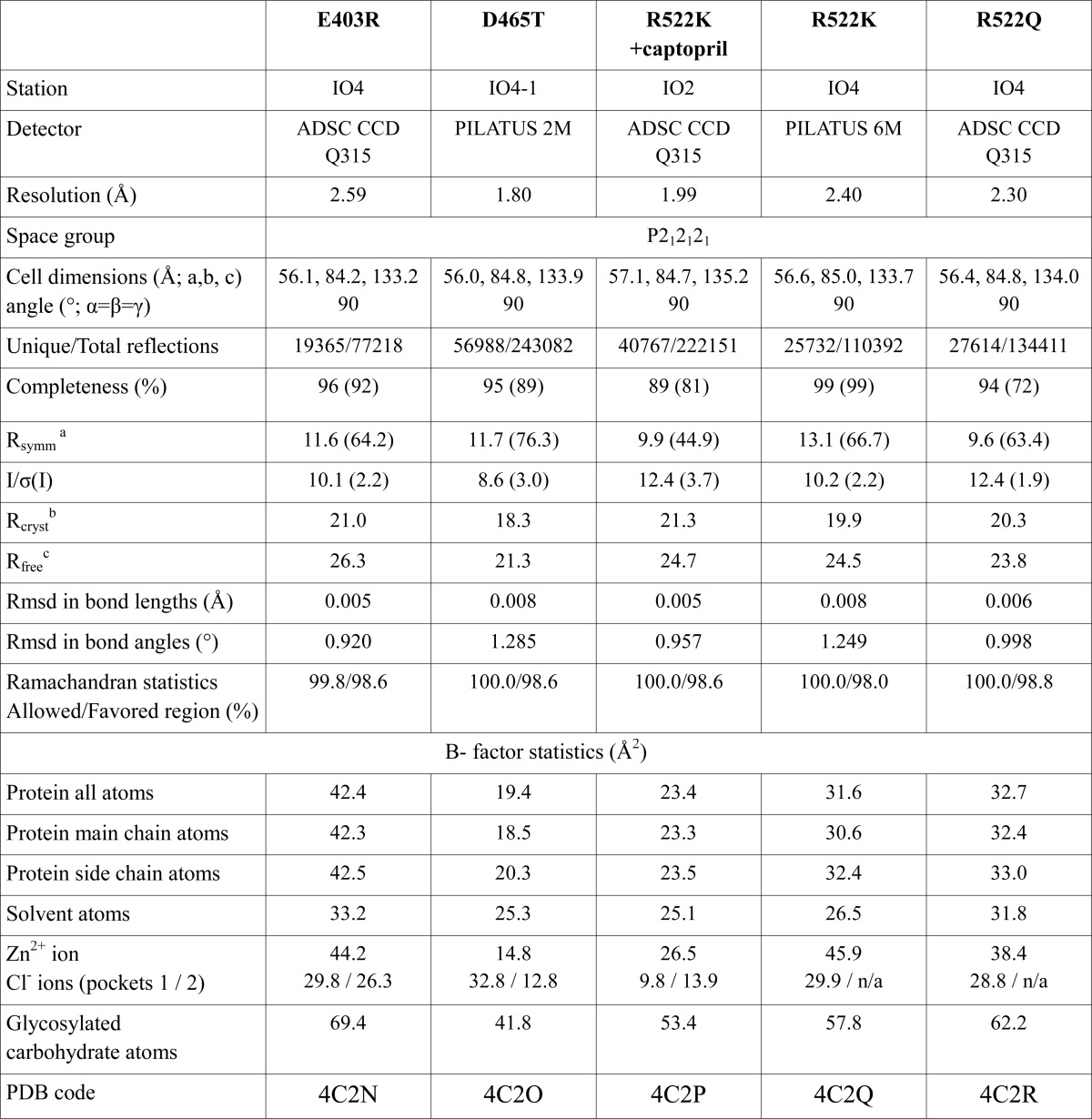
**Crystallographic statistics**

*^a^ R*_symm_ = Σ_*h*_Σ_*i*_[|*I*_*i*_(*h*) − 〈*I*(*h*)〉|/Σ_*h*_Σ*_i_ I_i_*(*h*)], where *I_i_* is the *i*th measurement, and 〈*I*(*h*)〉 is the weighted mean of all the measurements of *I*(*h*).

*^b^ R*_cryst_ = Σ*_h_*|*F_o_* − *F_c_*|/Σ*_h_F_o_*, where *F_o_* and *F_c_* are observed and calculated structure factor amplitudes of reflection *h*, respectively.

*^c^ R*_free_ is equal to *R*_cryst_ for a randomly selected 5% subset of reflections.

##### Chloride Titration Assays Using Hippuryl-l-His-Leu (HHL) and Z-FHL

Chloride titrations were performed using HHL and *Z*-FHL solutions containing varying amounts of NaCl (0–1 m) prepared using Milli-Q distilled H_2_O (chloride concentration <0.06 mm). All substrates used were HPLC-purified (>98% purity), so any chloride or chloride salts would elute with the breakthrough peak; hence, the effects on chloride concentrations are negligible. Assays were performed using a protocol adapted from that described previously (28), with the primary difference being use of a different buffer (50 mm TAPSO (pH 7.5)). Milliunits of ACE activity, with 1 unit defined as 1 nmol of HL produced/min/ml (min^−1^·ml^−1^) at 37 °C in assay buffer, were calculated, divided by the total number of μg in the reaction volume, and expressed as specific activity (milliunits·μg^−1^). Substrate concentrations used were ≥5 times *K_m_*, and the total percentage hydrolysis in each reaction was kept below 20% in order to approximate first order rates (*k*_cat_/*K_m_*). The measure of chloride binding (*K_d_*_(app)_) was calculated from activation plots of specific activity *versus* the logarithm of chloride concentration, with the resultant curves analyzed via non-linear regression using a sigmoidal dose-response curve with the formula,

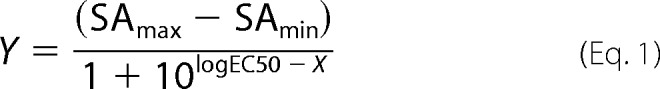
 where SA_min_ is the specific activity in the absence of NaCl, SA_max_ is the maximum specific activity upon titration with chloride, *X* is the logarithm of chloride concentration, *Y* is the response (specific activity), and EC_50_ is the *X* value when the response is halfway between SA_max_ and SA_min_.

##### Isothermal Titration Calorimetry Sample Preparation

Enzyme samples of high concentration (>10 μm) were dialyzed extensively against 3× 1 liter of reaction buffer, which consisted of 50 mm TAPSO buffer (pH 7.5), 10 μm ZnSO_4_, and either 0, 20, or 300 mm NaCl.

Substrate/inhibitor was prepared by either dissolution directly into dialysate, to the desired concentration (for HHL, Z-FHL, and lisinopril), or by equilibrating against a 1-ml G10 Sephadex desalting column (for angiotensin I). Angiotensin I (Asp-Arg-Val-Tyr-Ile-His-Pro-Phe-His-Leu) was dissolved in dialysate at high concentration then passed through a 1-ml G10 column (equilibrated with dialysate in order to reduce TFA concentrations) under gravity. 200-μl fractions were collected, and absorbance was measured at 275 nm. These were pooled, and the final concentration was determined spectrophotometrically via absorbance at 275 nm, using the empirically calculated extinction coefficient of 1280 m^−1^·cm^−1^.

##### Isothermal Titration Calorimetry Binding Assays

Assays were performed using an iTC_200_ microcalorimeter and according to the guidelines in the iTC_200_ microcalorimeter user manual provided by MicroCal. Buffer conditions for both enzyme and lisinopril consisted of 50 mm TAPSO buffer (pH 7.5), 10 μm ZnSO_4_, and either 0, 20, or 300 mm NaCl, prepared using Milli-Q distilled H_2_O (chloride concentration <0.06 mm). The reaction cell was maintained at 20 °C, with the lisinopril concentration in the syringe (800–1400 μm) at 10 times the enzyme concentration in the cell (8–12 μm) to account for lisinopril dilution (∼6-fold) and ensure a complete 1:1 binding curve. Binding assays were performed with every preparation of enzyme used in the kinetic assays; this served as an active site titration to determine active enzyme concentration in order to improve the accuracy of the *k*_cat_ (equal to *V*_max_/[*E*]) values obtained in those experiments. The *K_d_*_(app)_, Δ*G*, Δ*H*, and Δ*S* values for lisinopril binding were calculated using Origin 7 with the iTC_200_ MicroCal Software Addon.

##### Isothermal Titration Calorimetry Kinetic Assays

All ITC experiments were performed using an iTC_200_ microcalorimeter (MicroCal LLC), with raw data either extracted for custom calculations using Microsoft Excel or analyzed using Origin 7 software with a proprietary MicroCal analysis module. Assays were performed at 37 °C using an adapted version of the progress curve method described by Stockbridge and Wolfenden (29), where the variation comprised the use of substrate concentrations of 3–5 times *K_m_*. The assay setup for the titration calorimeter involves a single injection of substrate into enzyme or enzyme into substrate and the continuous monitoring of thermal power as substrate is catalyzed to completion. A means of converting this thermal power into a reaction curve of product formed over time has been described previously (29). Given that heat released or absorbed by the enzyme reaction is directly proportional to the amount of substrate hydrolyzed, the amount of product formed over time can be calculated, and a progress curve can be generated. Integration of the total area under the curve represents the total heat turned over in catalyzing all of the substrate. Integrating the area between substrate injection (*t* = 0) and each time point (*t*) and then dividing by the total integrated area gives a percentage of the product formed at time *t*. Multiplying this fraction at each time point by the total substrate concentration at *t* = 0 (*S*_0_) relates it to molar concentration. The formula describing this relationship is as follows,

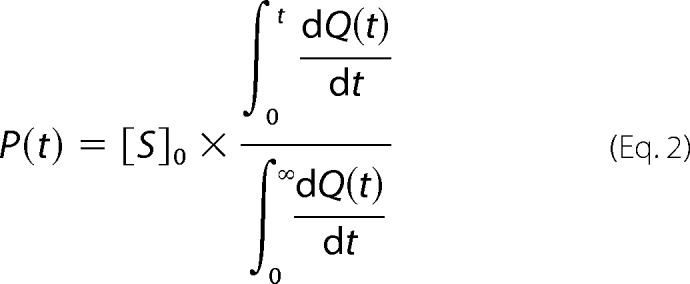
 with the resultant progress curve being a plot of *P*(*t*) (product formed) *versus t* (time).

Progress curves were generated and analyzed according to functions described by Golicnik (30). The temporal closed form solution of the Michaelis-Menten equation used is given by Equation 3,

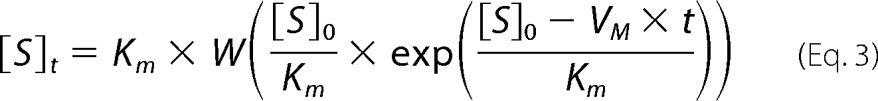
 where *W* is the Lambert *W*(*x*) function (31). Golicnik (30) evaluated a number of approximations of *W* and found one version that produced an acceptably low amount of systemic error when applied to Equation 3 in evaluating progress curves,

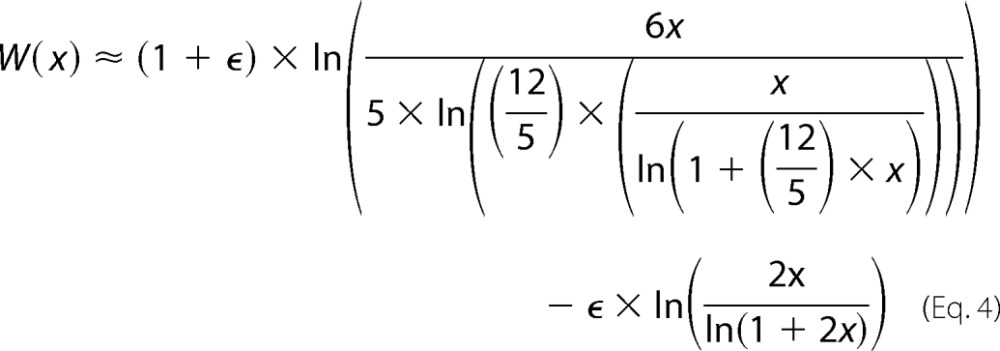
 where ϵ = 0.4586887. Progress curves were generated in Microsoft Excel using Equation 2 and analyzed using the combined Equations 3 and 4, which were written into Graphpad Prism 5 software according to instructions provided by Golicnik (30). Assays were performed in triplicate as a minimum, and all kinetic constants (*K_m_*, *k*_cat_, and *k*_cat_/*K_m_*) are reported as the mean of the three separate determinations along with the S.E. value.

The thermodynamic parameters (Δ*G*, Δ*H*, and Δ*S*) for the hydrolysis of substrates (HHL, Z-FHL, and angiotensin I) were determined using the kinetic data (*k*_cat_/*K_m_*) and the raw data from each of those assays. The Gibbs free energy (Δ*G*) was calculated by the formula Δ*G* = −*RT* ln(*k*_cat_/*K_m_*), with *R* being the gas constant (1.9858 cal·K^−1^·mol^−1^) and *T* being assay temperature (310 K). The apparent enthalpy (Δ*H*_app_) was determined for each assay via integration of the area under the curve using the following formula.

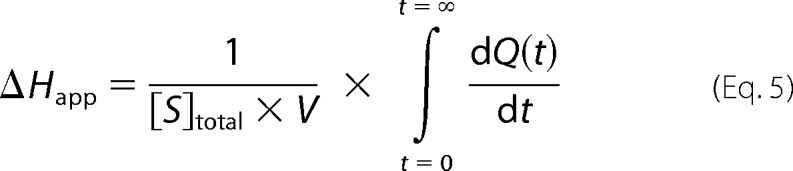
 To exclude the contribution of buffer to enthalpy, the intrinsic enthalpy (Δ*H*_int_) was calculated using the relationship, Δ*H*_app_ = Δ*H*_int_ + *n*_H_ × Δ*H*_ion_ (Δ*H*_ion_ for TAPSO buffer = 9.378 kcal/mol; *n*_H_ = −0.37 for all substrates). Incorporation of Δ*G* and Δ*H*_int_ into Δ*G* = Δ*H*_int_ − *T*Δ*S* allows calculation of −*T*Δ*S*, which is the entropy as a function of temperature. These values were calculated separately for each assay, which was done in triplicate as a minimum, with values shown as mean and S.E.

## RESULTS AND DISCUSSION

### 

#### 

##### Crystal Structures of Human C-domain sACE Mutants

**FIGURE 2.**
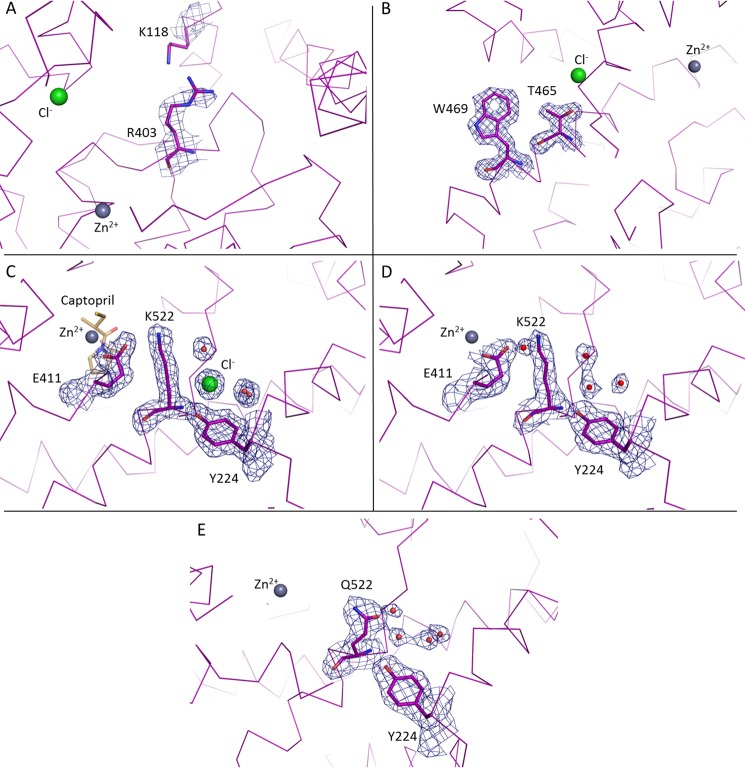
**Electron density map of mutated residues in C-domain ACE.** A weighted difference map was calculated with REFMAC5 and is displayed at 1σ level for E403R (*A*), D465T (*B*), R522K + captopril (*C*), R522K (*D*), and R522Q (*E*).

**FIGURE 3.**
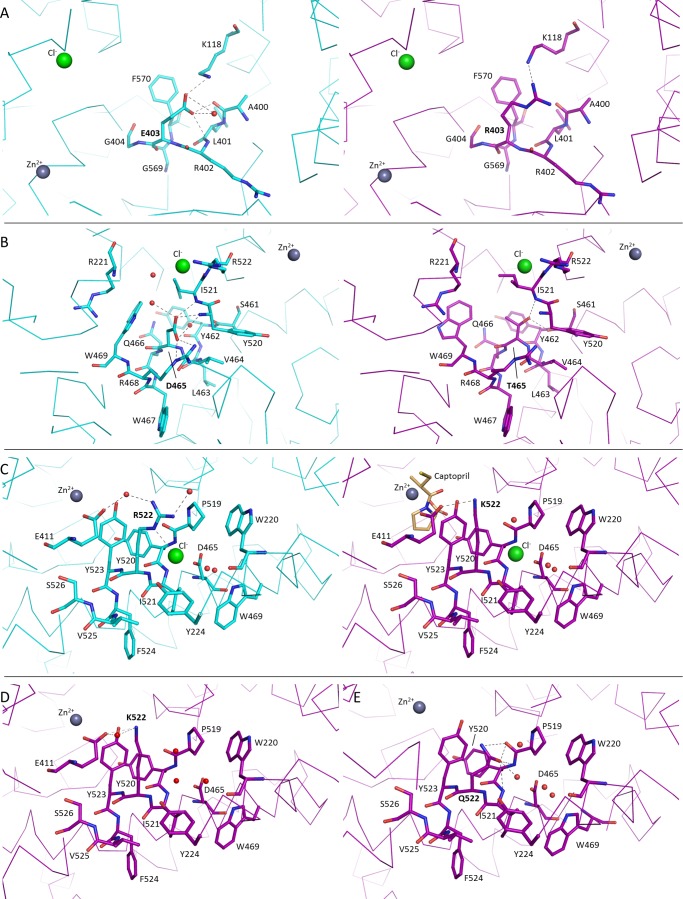
**Structural changes from mutations in C-domain ACE.** Comparison of local structural changes from mutations in C-domain ACE. Shown are native (*cyan*) and mutant (*magenta*) structures where E403R (*A*), D465T (*B*), R522K + captopril (*C*), R522K (*D*), and R522Q (*E*) are represented. Chloride and zinc ions are shown as *spheres* (*green* and *gray*, respectively), and water molecules are shown in *red*. Possible hydrogen bonds are shown as *dashed lines*.

Human C-domain mutants E403R, D465T, R522K, and R522Q were crystallized and data were collected at 2.59, 1.80, 1.99, and 2.30 Å resolution, respectively (Fig. 1 and Table 2). An unambiguous difference density map confirmed the mutations at the expected sites (Fig. 2). None of the mutations created caused any major conformational change in the overall architecture of the protein, with the root mean square deviation over an all-atom superposition being ≤0.4 Å for each of the mutants when compared with the C-domain (Fig. 1*B*). The consequences appeared to be localized around the mutated sites, which were designed to investigate the role of these specific residues in the chloride-dependent enzymatic activity. Mutations R522K (ligand-free) and R522Q showed the loss of the chloride ion in proximity to the active site (Fig. 3).

The E403R mutation is in a highly accessible solvent area, and thus the longer side chain presented lower stability. Furthermore, whereas Glu^403^ made a salt bridge with Lys^118^, mutation to Arg^403^ resulted in local disorder with a higher *B* factor seen for Lys^118^. The side chain of Lys^118^ was refined in this position as in the C-domain (Fig. 3*A*) but is expected to be very mobile, as illustrated by the lack of electron density (Fig. 2*A* and Table 3). E403R also presented less contact with the surrounding residues by the loss of several hydrogen bonds with Ala^400^, Leu^401^, and Arg^402^. No water molecule was seen interacting with the mutated residue despite its solvent accessibility, and overall, no conformational change of the main carbon chain was visible.

**TABLE 3:**
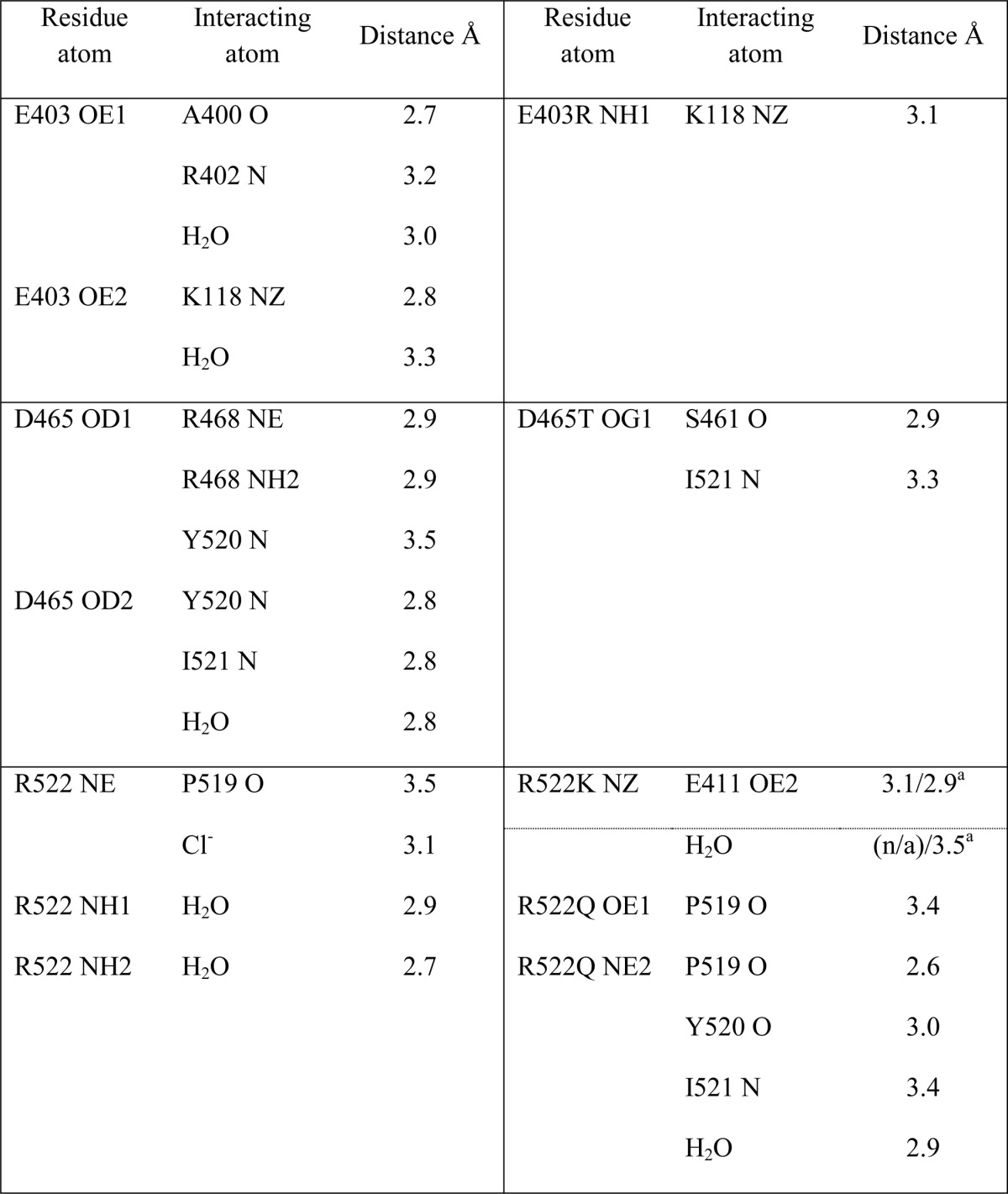
**Side chain hydrogen interactions in mutated residues**

*^a^* R522K structure with and without captopril, respectively.

A high resolution data set was collected for the D465T mutant. The native Asp^465^ side chain is stabilized by a salt bridge with Arg^468^ as well as hydrogen bonds with the main chain of Tyr^520^ and Ile^521^ (Fig. 3*B* and Table 3). Although it did not disrupt the local secondary α-helical structure, mutation to Thr^465^ resulted in reorientation of the side chain with a 60° rotation that allows it to interact with Ser^461^ and Ile^521^, thus losing the interaction with Arg^468^. Interestingly, this also led to a change in rotamer conformation of Trp^469^, which went from a water-mediated interaction with Asp^465^ to a hydrogen bond with Arg^221^. This created a new pocket that permitted the presence of a malonate molecule (a component of the crystallization buffer; data not shown). The malonic acid is coordinating with the same Arg^221^ on one side and mimicking the interactions previously made by Asp^465^ on the other side, including the salt bridge with Arg^468^ and hydrogen bonds with Tyr^520^ and Ile^521^. Noticeably, loop Ser^434^–Ser^439^ was visible for the first time in a C-domain crystal structure (Fig. 1*B*) with clear electron density observed for this flexible loop region.

The chloride ion is directly coordinated by Arg^522^ in the native C-domain, along with Tyr^224^ and a water-mediated interaction to Trp^220^ (Fig. 3*C*). The R522K mutant was crystallized with and without captopril (Fig. 2, *C* and *D*, respectively). Whereas the Cl^−^ ion is not visible in the ligand-free structure, the ion was clearly visible in the bound complex. There is no evidence from the electron density map of the R522K·captopril structure that the chloride is linked to Lys^522^ (Fig. 2*C*) with the amino group located more than 4 Å away from a water in the ion's hydration shell. The chloride ion is coordinated by the hydroxyl group of Tyr^224^ and the surrounding water molecules linked to Trp^220^, Trp^469^, and Asp^465^. Importantly, Lys^522^ now forms a salt bridge with Glu^411^, which is part of the zinc coordination motif and thus involved in the enzyme catalytic activity. Lys^522^ is also within the distance of a cation-π interaction with Tyr^523^, thus further directing the side chain away from the Cl^−^ ion. It should be added that captopril is present at the active site in the same position as that of the wild-type C-domain·captopril complex structure (32). Although its presence did not disturb the catalytic pocket, the ligand interacts with Tyr^523^ through a hydrogen bond and π stacking of its proline group with the phenol of Tyr^523^. In the ligand-free structure, the chloride ion is replaced by a water molecule within coordinating distance of Tyr^224^ (Fig. 3*D*). The position of the residues within the chloride pocket remains unchanged, and Lys^522^ is therefore making interactions similar to those described for the ligand-bound structure. An additional water-mediated contact was seen with Glu^411^, reinforcing the link with the catalytic pocket.

Mutation R522Q also resulted in the loss of the chloride ion (Fig. 2*E*), with Gln^522^ making strong hydrogen bonds with the backbone of residues Pro^519^, Tyr^520^, and Ile^521^ (Fig. 3*E* and Table 3) and a weak cation-π interaction with Tyr^523^. In place of the Cl^−^ is now a water molecule stabilized by Tyr^224^ and a network of other waters themselves linked to Asp^465^ and Trp^469^. Furthermore, a sulfate ion was present in the active site, making direct coordination with the zinc ion and surrounding residues of the S1 subsite, including Tyr^523^.

##### Effect of Chloride on Energetic Contributions in ACE Binding and Catalysis

Mutant C-domain constructs were characterized using the substrates HHL, Z-FHL, and AngI in the absence and presence of 20 mm NaCl. Kinetic constants and thermodynamic parameters are displayed in Tables 4 and 5, respectively.

**TABLE 4:** **Kinetic parameters for the cleavage of HHL, Z-FHL, and angiotensin I by ACE constructs** Shown is cleavage of HHL, Z-FHL, and angiotensin I by the C-domain, the N-domain, and a number of C-domain mutant constructs. Constants were determined using an ITC assay at 0 and 20 mm NaCl (including 300 mm for the C-domain with HHL) in 50 mm TAPSO (pH 7.5) and 10 μm ZnSO_4_. Values are shown as the mean of three separate determinations of *K_m_*, *k*_cat_, and *k*_cat_/*K_m_* along with S.E.

Construct	Substrate	[NaCl]	*K_m_*	*k*_cat_	*k*_cat_/*K_m_*
		*mm*	*mm*	*s*^−*1*^	*mm*^−*1*^ *s*^−*1*^
N-domain	HHL	0	3.910 ± 0.060	14.15 ± 0.53	3.63 ± 0.14
		20	0.720 ± 0.020	38.84 ± 0.13	53.97 ± 0.15
	Z-FHL	0	1.360 ± 0.010	73.38 ± 0.62	54.10 ± 0.47
		20	0.520 ± 0.010	509.50 ± 15.61	991.25 ± 31.06
	Angiotensin I	0	0.520 ± 0.010	4.04 ± 0.06	36.23 ± 0.47
		20	0.077 ± 0.006	24.98 ± 1.59	325.40 ± 20.54
C-domain	HHL	0	1.960 ± 0.020	3.26 ± 0.46	1.66 ± 0.24
		20	1.650 ± 0.030	127.00 ± 1.78	77.15 ± 1.03
		300	0.779 ± 0.008	606.00 ± 21.86	777.50 ± 28.11
	Z-FHL	0	0.580 ± 0.010	80.25 ± 1.20	140.00 ± 2.13
		20	0.120 ± 0.010	282.00 ± 11.16	2372.50 ± 93.67
	Angiotensin I	0	0.049 ± 0.001	1.70 ± 0.11	35.28 ± 2.11
		20	0.036 ± 0.001	7.38 ± 0.05	204.67 ± 1.21
R186H	HHL	0	2.740 ± 0.030	2.09 ± 0.08	0.77 ± 0.03
		20	2.060 ± 0.020	122.00 ± 1.16	59.27 ± 0.62
	Z-FHL	0	1.000 ± 0.040	76.24 ± 2.94	76.44 ± 2.94
		20	0.150 ± 0.010	467.34 ± 36.45	3193.34 ± 247.68
	Angiotensin I	0	0.085 ± 0.003	1.52 ± 0.03	17.93 ± 0.29
		20	0.095 ± 0.003	7.83 ± 0.19	82.65 ± 2.02
E403R	HHL	0	4.360 ± 0.050	20.48 ± 0.43	4.70 ± 0.10
		20	1.540 ± 0.040	459.00 ± 24.12	298.67 ± 15.90
	Z-FHL	0	0.530 ± 0.010	159.34 ± 2.91	304.34 ± 5.21
		20	0.540 ± 0.010	929.34 ± 14.77	1736.67 ± 28.49
	Angiotensin I	0	0.069 ± 0.003	3.96 ± 0.28	57.46 ± 4.07
		20	0.061 ± 0.004	9.08 ± 0.41	123.00 ± 5.42
R522Q	HHL	0	2.820 ± 0.020	6.18 ± 0.17	2.20 ± 0.06
		20	1.680 ± 0.010	4.80 ± 0.18	2.87 ± 0.11
	Z-FHL	0	0.110 ± 0.010	92.24 ± 2.16	860.34 ± 20.01
		20	0.200 ± 0.010	124.67 ± 9.36	653.34 ± 47.73
	Angiotensin I	0	0.092 ± 0.003	12.98 ± 0.29	141.75 ± 3.20
		20	0.077 ± 0.003	13.75 ± 0.84	179.50 ± 11.14
R522K	HHL	0	3.720 ± 0.020	5.31 ± 0.19	1.43 ± 0.06
		20	2.250 ± 0.020	2.03 ± 0.05	0.91 ± 0.02
	Z-FHL	0	0.570 ± 0.010	126.00 ± 1.00	223.25 ± 1.94
		20	0.880 ± 0.020	193.50 ± 5.13	220.75 ± 5.97
	Angiotensin I	0	0.130 ± 0.002	0.83 ± 0.03	6.37 ± 0.21
		20	0.119 ± 0.002	3.79 ± 0.07	32.05 ± 0.53

**TABLE 5:** **Thermodynamic parameters for the cleavage of HHL, Z-FHL and angiotensin I by ACE constructs** Shown are thermodynamic parameters associated with the cleavage of HHL, Z-FHL, and angiotensin I by the C-domain, N-domain, and a number of C-domain mutant constructs. Values were determined at 0 and 20 mm NaCl (including 300 mm for the C-domain with HHL). Values are shown as the mean of three separate determinations of Δ*G*, Δ*H*, and −*T*Δ*S* along with S.E.

Construct	Substrate	[NaCl]	Δ*H*_int_	Δ*G*	−*T*Δ*S*
		*mm*	*kcal mol*^−*1*^	*kcal mol*^−*1*^	*kcal K*^−*1*^ *mol*^−*1*^
N-domain	HHL	0	2.48 ± 0.03	−5.04 ± 0.02	−7.52 ± 0.05
		20	2.59 ± 0.01	−6.93 ± 0.00	−9.52 ± 0.01
	Z-FHL	0	2.25 ± 0.01	−6.71 ± 0.01	−8.96 ± 0.01
		20	2.80 ± 0.06	−8.50 ± 0.02	−11.29 ± 0.08
	Angiotensin I	0	2.80 ± 0.06	−6.46 ± 0.01	−11.29 ± 0.08
		20	2.71 ± 0.10	−7.81 ± 0.05	−10.52 ± 0.10
C-domain	HHL	0	2.37 ± 0.07	−4.19 ± 0.01	−6.56 ± 0.07
		20	2.73 ± 0.03	−6.93 ± 0.01	−9.66 ± 0.03
		300	2.60 ± 0.01	−8.33 ± 0.01	−10.93 ± 0.01
	Z-FHL	0	2.27 ± 0.03	−7.30 ± 0.01	−9.57 ± 0.04
		20	2.70 ± 0.04	−9.06 ± 0.02	−11.80 ± 0.03
	Angiotensin I	0	2.16 ± 0.07	−6.44 ± 0.04	−8.60 ± 0.10
		20	2.19 ± 0.04	−7.53 ± 0.00	−9.72 ± 0.04
R186H	HHL	0	2.25 ± 0.01	−3.75 ± 0.01	−6.00 ± 0.01
		20	2.72 ± 0.02	−6.77 ± 0.01	−9.49 ± 0.02
	Z-FHL	0	2.53 ± 0.06	−6.92 ± 0.02	−9.45 ± 0.08
		20	2.74 ± 0.08	−9.22 ± 0.05	−11.95 ± 0.07
	Angiotensin I	0	2.27 ± 0.04	−6.03 ± 0.01	−8.30 ± 0.03
		20	2.19 ± 0.01	−6.97 ± 0.02	−9.16 ± 0.02
E403R	HHL	0	2.62 ± 0.01	−5.21 ± 0.01	−7.82 ± 0.02
		20	2.93 ± 0.05	−7.76 ± 0.03	−10.69 ± 0.04
	Z-FHL	0	2.41 ± 0.03	−7.77 ± 0.01	−10.18 ± 0.04
		20	2.83 ± 0.10	−8.85 ± 0.01	−11.67 ± 0.11
	Angiotensin I	0	2.63 ± 0.05	−6.73 ± 0.05	−9.35 ± 0.10
		20	2.75 ± 0.11	−7.21 ± 0.03	−9.97 ± 0.10
R522Q	HHL	0	2.48 ± 0.03	−4.46 ± 0.02	−6.94 ± 0.05
		20	2.65 ± 0.16	−4.90 ± 0.02	−7.54 ± 0.18
	Z-FHL	0	2.38 ± 0.05	−8.41 ± 0.02	−10.79 ± 0.06
		20	2.51 ± 0.06	−8.24 ± 0.05	−10.75 ± 0.01
	Angiotensin I	0	2.28 ± 0.01	−7.30 ± 0.01	−9.59 ± 0.02
		20	2.36 ± 0.11	−7.45 ± 0.04	−9.80 ± 0.15
R522K	HHL	0	2.29 ± 0.05	−4.47 ± 0.02	−6.76 ± 0.07
		20	2.24 ± 0.04	−4.46 ± 0.01	−6.69 ± 0.04
	Z-FHL	0	2.22 ± 0.02	−7.58 ± 0.01	−9.81 ± 0.03
		20	2.78 ± 0.04	−7.58 ± 0.02	−10.36 ± 0.05
	Angiotensin I	0	2.21 ± 0.04	−5.39 ± 0.02	−7.24 ± 0.02
		20	1.85 ± 0.00	−6.39 ± 0.01	−8.60 ± 0.04

**FIGURE 4.**
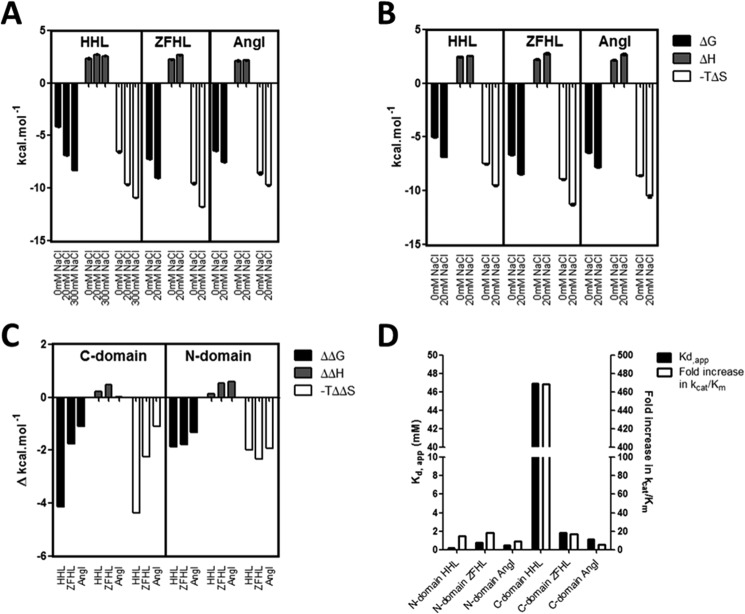
**Effect of chloride concentration on thermodynamic parameters associated with ACE hydrolysis.** Shown is the thermodynamic signature for the hydrolysis of HHL, Z-FHL, and angiotensin I at 0 and 20 mm NaCl by the C-domain (*A*) and N-domain (*B*). The values for the C-domain with HHL at 300 mm NaCl are included because this is the concentration of maximal activity and chloride saturation for this substrate and domain (whereas 20 mm is the maximum for the other values). *C*, the ΔΔ*G*, ΔΔ*H*, and −*T*ΔΔ*S* values for the C-domain and N-domain represent the difference in Δ*G*, Δ*H*, and −*T*Δ*S* between 0 and 20 mm (between 0 and 300 mm for HHL with the C-domain). *D*, relative *K_d_*_(app)_ values (*left y* axis) and -fold increase in *k*_cat_/*K_m_* from 0 mm to maximal activity (*right y* axis) for C- and N-domains with HHL, Z-FHL, and AngI. *Error bars*, S.E.

In evaluating the effect of chloride on the thermodynamic profile of the C- and N-domains, the thermodynamic parameters were determined in the presence and absence of chloride (Fig. 4, *A* and *B*). Overall, the enthalpy (Δ*H*) is positive and very similar between the N- and C-domains as well as between substrates for both domains, indicating that the enthalpic contributions are very similar between the domains. What is also evident for all substrates with both domains is the larger contribution to Δ*G* by entropy (−*T*Δ*S*) than enthalpy (Δ*H*), showing that binding is most likely driven by hydrophobic interactions, with desolvation of substrate and structural changes being possible contributions.

There was a significant decrease in ΔG, associated with reduced catalysis, in the absence of chloride for C- and N-domains using AngI, HHL, and Z-FHL (Fig. 4, *A* and *B*). For all three substrates, there were negligible changes in the Δ*H* values, suggesting that increased activity in the presence of chloride is entropically driven. The fairly consistent Δ*H*, with and without chloride, between substrates suggests that the main interactions that form part of the catalytic mechanism are preserved, which is consistent with the observation that catalysis does occur in the absence of chloride.

In order to better evaluate the chloride-dependant shifts in thermodynamic data, the changes in Δ*G*, Δ*H*, and −*T*Δ*S* (ΔΔ*G*, ΔΔ*H*, and −*T*ΔΔ*S*) between 0 mm NaCl and the concentration of maximal activity (300 mm for HHL, 20 mm NaCl for Z-FHL and AngI) were calculated for both domains (Fig. 4*C*). For the C-domain, the greatest change in Δ*G* is observed with HHL, with reduced change seen for Z-FHL and even less for AngI. These changes in free energy are almost completely entropically-driven, as evidenced by the −*T*Δ*S* values showing an almost identical trend. There is also almost no change in the enthalpy for AngI, with minor increases for HHL (0.24 kcal/mol) and Z-FHL approaching 0.5 kcal/mol (0.48 kcal/mol). The cleavage of a peptide bond would presumably lead to a lowering of enthalpy by 2–5 kcal/mol. However, it can be assumed that the conformational changes suggested by the negative −*T*ΔΔ*S* values would result in the formation of sufficient interactions, presumably stabilizing in nature, to counterbalance the effect of peptide bond cleavage and result in an overall slightly positive ΔΔ*H*. The entropy-linked increase in enthalpy is probably the result of hydrophobic forces, either via desolvation of hydrophobic groups upon binding of substrate or via the formation of new bonds with a structural change. By contrast, the corresponding ΔΔ*G*, ΔΔ*H*, and −*T*ΔΔ*S* values for the N-domain present far less variation between the different substrates. The ΔΔ*G* and −*T*ΔΔ*S* for HHL are considerably less negative (∼2-fold), whereas, in comparison, those for Z-FHL are relatively unchanged, with the AngI values being slightly more negative than those for the C-domain. The larger entropic shift for AngI hydrolysis with the N-domain is driven by a ∼0.5 kcal/mol increase in the enthalpy, which could be a loss of specific interactions due to variability of interactions with AngI between the two domains.

The largest variation in energetic contributions between domains is seen with HHL, suggesting that the difference in substrate composition, and hence interactions, compared with Z-FHL and AngI could be responsible for the observed shift. To highlight this variation, the *K_d_*_(app)_ values were compared with the -fold increase in *k*_cat_/*K_m_* between 0 mm NaCl and maximal activity for HHL, Z-FHL, and AngI for both domains (Fig. 4*D*). The *K_d_*_(app)_ and -fold increase in activity for the C- and N-domains are considerably higher for HHL. This indicates that the chloride dependence mechanisms of the C- and N-domains are most pronounced with the shorter HHL substrate and modulated by the increased length of the Z-FHL and AngI substrates.

##### Chloride 1 Pocket

**FIGURE 5.**
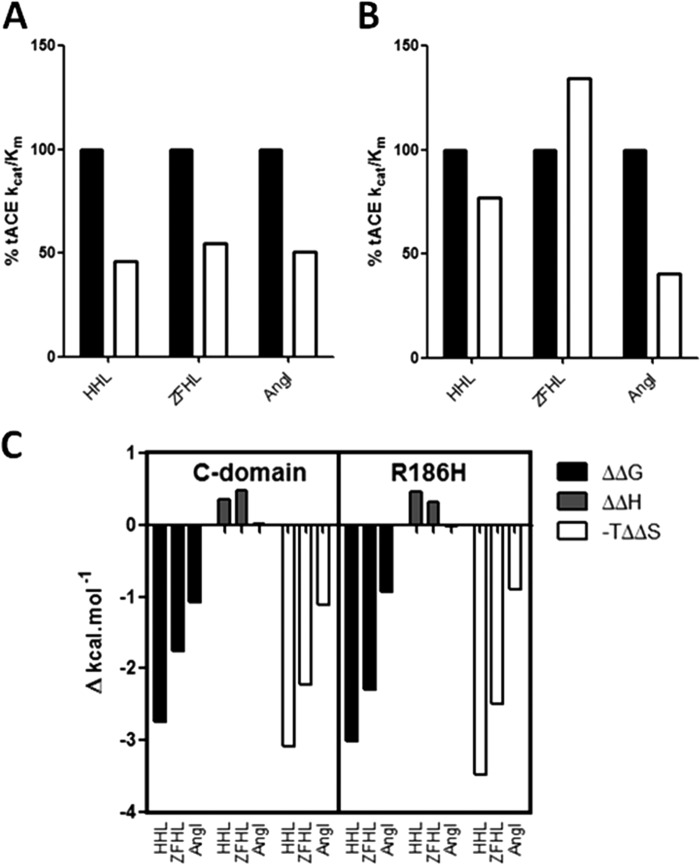
**Kinetic and thermodynamic comparisons for R186H.** The *k*_cat_/*K_m_* for the C-domain (*black bars*) and R186H (*white bars*) taken as the percentage of the *k*_cat_/*K_m_* for C-domain at 0 mm (*A*) and 20 mm (*B*) NaCl. *C*, the ΔΔ*G*, ΔΔ*H*, and −*T*ΔΔ*S* values for C-domain and R186H represent the difference in Δ*G*, Δ*H*, and −*T*Δ*S* between 0 and 20 mm NaCl.

The chloride 1 pocket was proposed to be involved in C-terminal stabilization of substrates (10, 33), and amino acid mutations in this pocket abrogated chloride activation with AngI (34). To further investigate the role of the chloride 1 pocket and how it might affect interactions in the S1′ and S2′ subsites, the R186H mutation was evaluated. In this mutation, the chloride-coordinating Arg in the C-domain was converted to a His, the corresponding residue in the N-domain and the only difference in the respective chloride 1 pockets between the two domains. The initial chloride titration results indicated that R186H produced no significant change in chloride binding for both HHL and Z-FHL. A comparison of the relative levels of enzymatic activity for R186H with the C-domain and for HHL, Z-FHL, and AngI at 0 and 20 mm NaCl is shown in Fig. 5, *A* and *B*. At 0 mm NaCl, all substrates show an approximate halving of activity relative to the C-domain (Fig. 5*A*), which can be attributed to increased *K_m_* values (Table 4). In the absence of free chloride, this indicates that the chloride 1 pocket probably affects substrate binding. At 20 mm chloride, the AngI *k*_cat_/*K_m_* for R186H relative to the C-domain is 40.4% (Fig. 5*B*), which compares favorably with results previously reported for a R186Q mutant (34). These variations in *k*_cat_/*K_m_* for AngI are due to the *K_m_* for R186H (0.095 mm) being almost 3-fold higher than for the C-domain (0.036 mm), with very little change in *k*_cat_ (7.83 and 7.38 s^−1^, respectively). This is in contrast to Z-FHL, where the change is due to an almost 2-fold increase in *k*_cat_ from the C-domain (282.0 s^−1^) to R186H (509.5 s^−1^), with a concomitant minor increase in *K_m_* (0.120 and 0.150 mm, respectively). HHL hydrolysis showed a similar pattern to AngI, with an increased *K_m_* from the C-domain to R186H, yet a change in *k*_cat_ with Z-FHL is observed, suggesting that interactions in the chloride 1 pocket play a role in mediating both substrate binding and catalysis.

Supporting this, a large change in binding affinity with lisinopril for R186H relative to the C-domain is also observed, with the *K_d_* for R186H (132 ± 6.2 nm) being ∼3-fold higher than for the C-domain (45.5 ± 4.24 nm) at 0 mm NaCl, as well as ∼3-fold lower at 20 mm NaCl (4.42 ± 0.5 and 13.5 ± 2.06, respectively) (Table 6). This further indicates that chloride is modulating the conformation of the prime binding sites via the chloride 1 pocket in the C-domain. When comparing the enthalpic change for the C-domain with the R186H mutant, there is some difference (10–50%) upon chloride binding (ΔΔ*H*) for the substrates yet not more than 11% change in the overall Δ*H* values (Fig. 5*C*). This suggests that there is no significant variation in interactions and that the effect is probably due to minor structural changes. The lack of a distinct effect on activity-associated chloride binding in the chloride 1 pocket of the R186H mutant supports the theory that the chloride 2 pocket is the main mechanistic effector of chloride dependence and that the chloride 1 pocket exerts its influence primarily via affinity modulation in the prime binding site. This also serves as a key differentiator in chloride-mediated substrate/inhibitor affinity between the domains.

**TABLE 6:** **Determination of lisinopril binding for all constructs** The *K_d_* values (nm) for lisinopril binding at 0 and 20 mm for the C-domain, the N-domain and a number of C-domain mutant constructs. Constants were determined using an ITC assay at 0 and 20 mm NaCl in 50 mm TAPSO (pH 7.5) and 10 μm ZnSO_4_. The S.D. value was calculated using the non-linear regression fitting function provided.

Construct	[NaCl]	*K_d_*
	*mm*	*nm*
N-domain	0	47.80 ± 4.94
	20	4.61 ± 0.63
C-domain	0	45.50 ± 4.24
	20	13.50 ± 2.06
R186H	0	132.00 ± 6.72
	20	4.42 ± 0.50
E403R	0	2.67 ± 0.31
	20	6.76 ± 0.65
R522Q	0	22.00 ± 3.31
	20	15.90 ± 2.09
R522K	0	35.30 ± 2.85
	20	26.30 ± 1.86

In the absence of a crystal structure of the R186H mutant, it is difficult to make any prediction as to what may be occurring and whether chloride would be present in the pocket. In the absence of chloride, the histidine of R186H could potentially form stacking interactions with Trp^182^ as (N-domain) His^164^ does with Trp^160^ and with Arg^489^ showing similar dual coordination of the Trp^279^ carbonyl. However, this would not explain why R186Q (34) abrogated chloride dependence.

##### Chloride 2 Pocket

Although the nature of the prime binding dipeptide and interactions within the chloride 1 pocket may partially explain the varying levels of overall activity between domains, it does not adequately describe how these interactions affect binding of chloride in the chloride 2 pocket, which has been shown to be mechanistically important (35).

In evaluating the crystal structures of the C-domain and the D465T mutant, extensive hydrogen bonding between the Asp^465^ carboxylate and primary amides of both Tyr^520^ and Ile^521^ is seen (Fig. 3*B*). Tyr^520^, Ile^521^, Arg^522^, and Tyr^523^ are the first four residues on the C-terminal end of the α23 helix (Tyr^520^–Ala^541^), with Pro^519^ representing the end of the helix and the beginning of a highly variable loop. However, this region of the α23 helix is within subdomain II of both domains, a region predicted to have little major structural variation relative to the proposed hinge region that is part of subdomain I (8). The C-terminal end of α23 is the main point of contact with α20, supporting a structural role for Asp^465^. Furthermore, in the C-domain, Asp^465^ hydrogen-bonds with Arg^468^, which interacts strongly with the variable loop via hydrogen bonds with backbone primary carbonyls of His^513^ and Val^518^. In the N-domain, Asp^443^ (which corresponds to C-domain Asp^465^) does not interact with Arg^446^ (Arg^468^ in the C-domain), but Arg^446^ does hydrogen-bond with Thr^496^, which corresponds to Val^518^ in the C-domain. Both Val^518^, in the S1 subpocket, and His^513^, which interacts with Tyr^523^ (10), are important residues for peptide binding that reside on this variable loop (36). Given that Asp^465^ is one of very few contacts between helices α23 and α20, its role is most likely in stabilization of the C-terminal end of helix α23.

The chloride titration data determined for HHL and Z-FHL using the D465T mutant (Table 7) support this assertion. The maximum specific activity in the presence of chloride (SA_max_) and the activity at 0 mm NaCl as a percentage of maximal activity (%SA_max_) for D465T with both HHL and Z-FHL were lower than those of the C-domain. An increase in *K_d_*_(app)_ for Z-FHL and a decrease for HHL with D465T was also observed. The location of Asp^465^ on the side of the chloride 2 pocket opposite from the proposed chloride channel area precludes any direct interaction with the chloride ion and involvement in the chloride channel mechanism. These results may be explained by the fact that Asp^465^ stabilizes the C-terminal end of helix α23 and the variable loop via hydrogen bonding with the primary amides of Tyr^520^ and Ile^521^. The Thr in this position is capable of fewer potential hydrogen bonds (Fig. 3*B* and Table 3), resulting in more structural movement that affects the C-terminal carboxylate coordinating Tyr^520^ (lowering of SA_max_). Its effect on %SA_max_ and *K_d_*_(app)_ is probably due to structural destabilization, illustrated by the rearrangement of Trp^469^ (Fig. 3*B*), which suggests that structural rotation or movement around the C-terminal end of helix α23, which contains Arg^522^ and Tyr^523^, moderates not only activity but chloride affinity.

**TABLE 7:** **Effect of NaCl on the hydrolysis of HHL and Z-FHL synthetic peptides by ACE constructs** All values were obtained from chloride-titrated activity assays. Units for SA_min_ and SA_max_ are milliunits·μg^−1^, and *K_d_*_(app)_ values are in mm. Data shown represent the average of two independent determinations, each in triplicate. The approximation symbol indicates that accurate estimates of *K_d_*_(app)_ and SA_max_ could not be obtained because the chloride concentration range was insufficient for saturation to occur. SA_max_ for these values is activity at 500 and 50 mm NaCl for HHL and Z-FHL, respectively.

Construct	HHL				Z-FHL			
	*K_d_*_(app)_	SA_min_	SA_max_	SA_min_ % of SA_max_	*K_d_*_(app)_	SA_min_	SA_max_	SA_min_ % of SA_max_
	*mm*	*milliunits*/μ*g*	*milliunits*/μ*g*	%	*mm*	*milliunits*/μ*g*	*milliunits*/μ*g*	%
N-domain	0.2	10.5	24.2	43.5	0.8	36.5	206.7	17.6
C-domain	46.9	2.1	83.2	2.5	1.8	93.5	339.3	27.5
R186H	76.9	3.7	113.3	3.2	2.1	130.3	467.2	27.8
E403R	2.7	6.3	99.8	6.3	0.3	214.5	398.5	53.8
D465T	12.7	0.4	46.6	0.8	2.8	2.1	110.7	1.8
R522K	∼339.7	4.1	∼10.0	40.5	∼104.6	97.2	∼193.2	50.3
R522Q	∼417.8	4.6	∼11.2	40.8	∼231.9	161.9	∼218.0	74.3

Thus, the interaction between Arg^522^ and Tyr^523^ is likely to influence chloride activation. The crystal structure of native C-domain showed the possible cation-π bond between these two residues, whereas the structures of the two 522 mutants demonstrated little structural change apart from the loss of chloride coordination.

**FIGURE 6.**
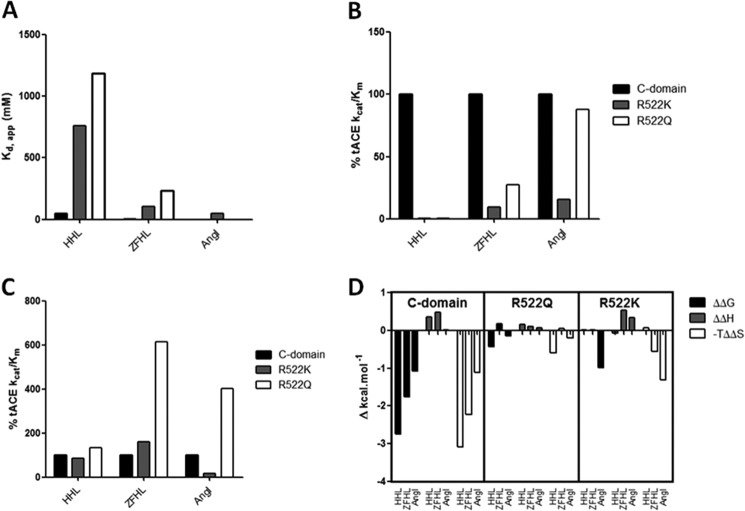
**Trends in chloride binding and activity for R522Q and R522K.** Shown is a graphical representation of chloride binding and kinetic values obtained for C-domain (*black bars*), R522K (*gray bars*), and R522Q (*white bars*) with HHL, Z-FHL, and AngI as substrates. *A*, the *K_d_*_(app)_ values (mm) for chloride binding. *, AngI values were estimated from the report of Liu *et al.* (35). The *k*_cat_/*K_m_* value was taken as the percentage of the *k*_cat_/*K_m_* for the C-domain at 0 mm (*B*) and 20 mm (*C*) NaCl. *D*, change in thermodynamic parameters for R522Q and R522K. The ΔΔ*G*, ΔΔ*H*, and −*T*ΔΔ*S* values for C-domain, R522Q, and R522K with HHL, Z-FHL, and AngI are shown and represent the difference in Δ*G*, Δ*H*, and −*T*Δ*S* between 0 and 20 mm NaCl.

Given these potential interactions, the kinetic data obtained for R522K and R522Q mutants relative to C-domain can be assessed. Comparing the *K_d_*_(app)_ values for HHL and Z-FHL (Fig. 6*A*), the largest increases relative to the C-domain are seen for HHL with R522K (7-fold) and R522Q (9-fold). A similar trend is seen with the *K_d_*_(app)_ values for Z-FHL and AngI (despite the lack of R552Q *K_d_*_(app)_) although not to the same degree as for HHL.

The replacement of Arg^522^ with a lysine or a glutamine causes a decrease in chloride affinity, in agreement with the structural data. Furthermore, R522K and R522Q follow the same *K_d_*_(app)_ trend as the C-domain (AngI < Z-FHL < HHL) although proportionally higher than the C-domain for each. This indicates that substrate interactions are somehow moderating access and binding of the chloride ion. This is confirmed by the presence of chloride in the pocket of R522K only when a ligand is bound at the active site (and captopril is a peptide mimetic). Substrate binding would cause local restructuring to interact with the catalytic site and thereby also close access to the chloride pocket. The sulfhydral group of captopril coordinates the zinc ion, thereby causing a loss of the hydrogen bond from Tyr^523^ seen in the tACE·lisinopril complex (32) and the transition state intermediate of the substrate. This could allow for more favorable interaction with the chloride ion.

Other interactions in the chloride 2 pocket are the salt bridge between Lys^522^ and Glu^411^ as well as the cation-π interaction of charged 522 residues with Tyr^523^. As would be expected with disruption of a key active site residue, the maximal *k*_cat_/*K_m_* values for R522K and R522Q are lower than that for the C-domain, with R522K lower than R522Q (Table 3). To better gauge the relative effects, the *k*_cat_/*K_m_* values for R522K and R522Q at 0 and 20 mm NaCl are represented as a percentage of the *k*_cat_/*K_m_* values for the C-domain (Fig. 6, *B* and *C*). At 0 mm NaCl, R522Q shows a marked increase in activity over both C-domain and R522K with Z-FHL and AngI (Fig. 6*B*), suggesting that additional interactions for the 522 position beyond chloride coordination are important. At 20 mm NaCl, there is an increase in activity for both R522K and R522Q with larger substrates that interact more tightly with the S1 and S2 active site pockets (Fig. 6*C*). Although R522K shows only a marginal increase over HHL (AngI (15.6%) > Z-FHL (9.3%) > HHL (0.1%)) relative to C-domain at 20 mm NaCl, there is a marked increase for R522Q (AngI (87.7%) > Z-FHL (27.5%) > HHL (0.36%)) to the point where R522Q activity is almost as high as C-domain activity with AngI. This would indicate that the bonding pattern is different between R522K and R522Q and that interactions of longer substrates have an effect on either the chloride 2 pocket or a key catalytic mechanism. To further probe this trend, the contributions of *K_m_* and *k*_cat_ to the catalytic efficiency of R522Q and R522K are evaluated. For HHL, the mechanistic lack of activation is demonstrated by the low variation in *K_m_* but marked differences in the *k*_cat_, where values are similar at 0 mm chloride and only C-domain showing an increase at 20 mm. Interestingly, R522Q shows a 5-fold lower *K_m_* for Z-FHL at 0 mm NaCl than C-domain, yet this trend is reversed at 20 mm, with the *K_m_* for R522Q being ∼2-fold higher than that for the C-domain. The data show that *K_m_* for R522K and R522Q with Z-FHL is higher at 20 mm than at 0 mm, a reversal of the general trend for the C-domain with all substrates. Both mutants show less than 2-fold increases in *k*_cat_ from 0 to 20 mm NaCl, *versus* the greater than 3-fold increase observed for the C-domain. Thus, the large decrease in activation for R522Q and R522K with Z-FHL is influenced by the increased *K_m_* as well as the reduced *k*_cat_ associated with the presence of chloride, in contrast to HHL, which is predominantly influenced by *k*_cat_. Interestingly, a similar lack of variance is seen in *K_m_* values for R522Q and R522K with AngI and greater variation in *k*_cat_. The increases in *K_m_* observed for all substrates with R522K and, to a lesser degree, R522Q indicate that coordination by chloride in this pocket affects substrate affinity as well as catalysis, suggesting some structural movement around helix α23. The most noticeable effect of the R522Q mutation is the greater *k*_cat_ values that increase with substrate length and are even higher than C-domain for AngI. Taking into account the structural data, it is likely that the lack of chloride coordination allows for more freedom of movement at the S1 recognition site, particularly for Tyr^523^, thus resulting in more efficient catalysis. It is therefore unlikely that the cation-π interaction is favored over the hydrogen bonds between Lys^522^ and Glu^411^ and between Gln^522^ and α23, respectively.

The changes in thermodynamic profiles in the presence of chloride for R522Q and R522K were evaluated (Fig. 6*D*). For R522Q, a reduced endothermic change in enthalpy is seen for HHL (0.165 kcal/mol) and ZHFL (0.105 kcal/mol) relative to the C-domain (0.36 and 0.48 kcal/mol, respectively), which would indicate the loss of an interaction(s) with the binding site. That the ΔΔ*H* values do not exceed the minimum hydrogen bonding energy (1 kcal/mol) might be due to differing hydration effects associated with the different residue counteracting the enthalpic change.

**FIGURE 7.**
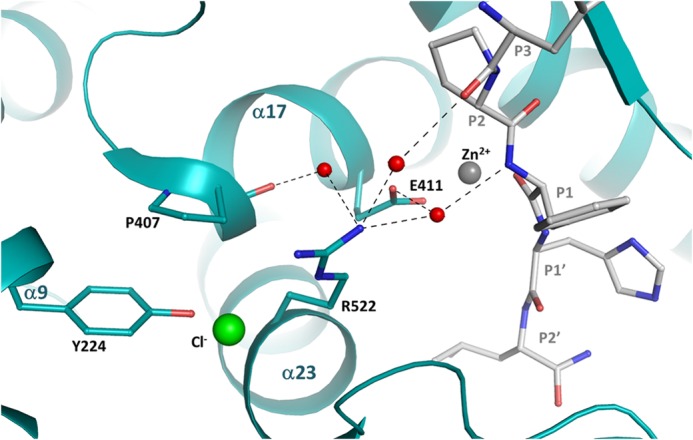
**Model of angiotensin I binding to C-domain ACE.** The structure of the C-domain ACE·Ang complex (Protein Data Bank code 4APH (36)) was used to model Angiotensin I before catalysis. The AngI peptide is represented in *gray*, chloride is shown in *green*, zinc ion is shown in *gray*, and water molecules are shown in *red*. The potential hydrogen bonds are shown in *dashed lines*. Residue 522 is presented in a *stick representation*.

For R522K, there is a different pattern with the enthalpy changes, with HHL (−0.07 kcal/mol) showing an exothermic shift relative to the C-domain (0.36 kcal/mol), AngI (0.35 kcal/mol) showing an endothermic shift relative to the C-domain (0.02 kcal/mol), and Z-FHL relatively unchanged. As with R522Q, an exothermic shift would suggest a bond breaking for HHL, which would be expected with the lack of direct chloride coordination. The lack of variation in enthalpy with Z-FHL for R522K is interesting, considering that there is no ΔΔ*G* and the *k*_cat_/*K_m_* is lower than C-domain and R522Q at both 0 and 20 mm NaCl. Given the assertions that Lys neither coordinates chloride nor cation-π-bonds with Tyr^523^, the interactions of Z-FHL in the S1 and S2 pockets may be compensating via minor structural shifts. The endothermic shift in enthalpy observed with AngI for R522K is accompanied by a relatively large shift in free energy (−0.99 kcal/mol) similar in magnitude to that in the C-domain (−1.09 kcal/mol), which actually results in a larger −*T*ΔΔ*S* value due to the more endothermic enthalpy shift. There is a 5-fold increase in *k*_cat_/*K_m_* from 0 to 20 mm NaCl, resulting in the observed ΔΔ*G*, although these values are considerably lower than in the C-domain. The modeling of AngI bound to C-domain (Fig. 7) based on the structure of the bound (cleaved) peptide complex structure (36) showed the possible interaction between Arg^522^ and the substrate through a number of water-mediated hydrogen bonds. Although it is unlikely that specific side chains are involved, the peptide backbone (positions P1 and P3) is within the distance (5.5 Å) of the Arg^522^ hydration shell. These important interactions may account for the chloride-mediated ΔΔ*G* as R522K and the catalytic site overall (through bonding with Glu^411^ and α17) lose the stability provided by the chloride ion.

Chloride coordination appears necessary to stabilize Arg^522^ toward the catalytic channel, thereby promoting catalysis of substrate. Substrate interactions within the S1/S2 pockets affect positioning of this complex and provide a rationale whereby substrate binding modulates chloride affinity and hence its own catalysis.

##### Chloride Channel

The E403R mutation was generated to investigate a proposed chloride channel, where Glu^403^ may act as an ionic gate mediating access of chloride to the chloride 2 pocket (37). For the E403R mutation, the Glu was converted to an Arg, corresponding to Arg^381^ in the N-domain (Table 1). The E403R mutation significantly reduced *K_d_*_(app)_ for chloride binding with both HHL and Z-FHL relative to the C-domain (Table 6). This effect was reversible in the N-domain when Arg^381^ was converted to a Glu, where the *K_d_*_(app)_ values showed 29- and 15-fold increases for HHL (4.7 mm) and Z-FHL (11.4 m) over the N-domain (0.2 and 0.8 mm, respectively). Furthermore, substrate interactions in the S2 subpocket where Glu^403^ resides might disrupt any ion gating, as evidenced by the large discrepancy in *K_d_*_(app)_ seen between HHL and Z-FHL for the C-domain, which was largely abolished with E403R.

**FIGURE 8.**
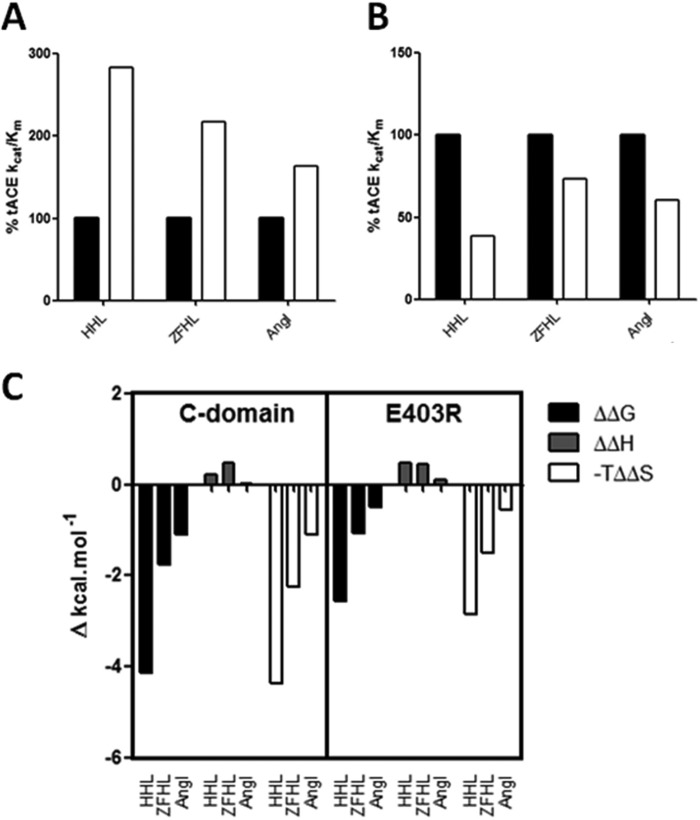
**Trends in chloride binding and activity for E403R.** Shown is a graphical representation of *k*_cat_/*K_m_* kinetic values obtained for the C-domain (*black bars*) and E403R (*white bars*) with HHL, Z-FHL, and AngI as substrates. The *k*_cat_/*K_m_* value was taken as the percentage of the *k*_cat_/*K_m_* for the C-domain at 0 mm (*A*) and 20 mm (*B*) NaCl. *C*, change in thermodynamic parameters for E403R. The ΔΔ*G*, ΔΔ*H*, and −*T*ΔΔ*S* values for the C-domain and E403R are shown and represent the difference in Δ*G*, Δ*H*, and −*T*Δ*S* between 0 and 20 mm NaCl (0 and 300 mm for HHL with C-domain).

**FIGURE 9.**
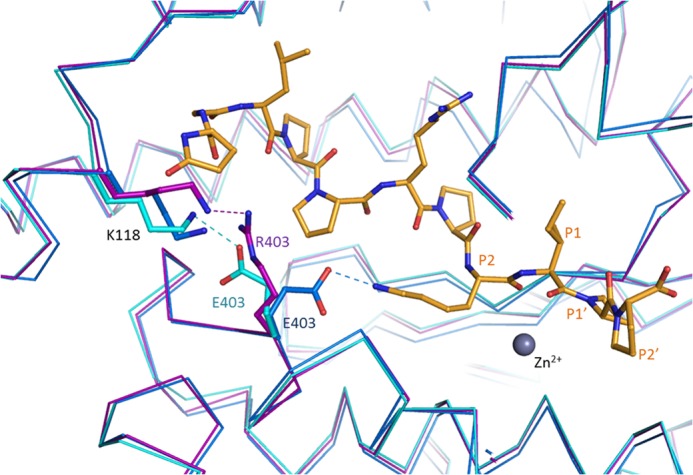
**Comparison of C-domain ACE bound to peptide inhibitor BPPb and the E403R mutant structures.** The structures of the C-domain ACE (*cyan*; Protein Data Bank code 4APJ (36)) and C-domain ACE·BPPb complex (*blue*; Protein Data Bank code 4APJ (36)) were superposed onto the C-domain E403R structure (*magenta*). The BPPb peptide is represented in *yellow*, and the zinc ion is shown in *gray* (not present in the BPPb complex structure). Interactions with position 403 are shown as *dashed lines* (*colors* correspond to the respective structures).

Interestingly, *K_d_*_(app)_ varies with different substrates, suggesting that chloride coordination is affected, but changes in overall activity and degrees of activation are also seen, thus highlighting further functions for Glu^403^. The E403R *k*_cat_/*K_m_* values are shown relative to the C-domain at 0 mm NaCl (Fig. 8*A*) and at maximal activity (Fig. 8*B*) for HHL, Z-FHL, and AngI. The *k*_cat_/*K_m_* values for E403R with all substrates at maximal activity are generally lower relative to the C-domain, whereas at 0 mm NaCl, they are all higher. This indicates that Glu^403^ affects activity as well as potentially mediating access to the chloride 2 pocket. Glu^403^ makes a salt bridge with Lys^118^ in the native structure (Fig. 9). Mutation of the Lys^118^-corresponding residues, Lys^154^ in rabbit testicular ACE (38) and Lys^694^ of rabbit lung ACE (39), drastically reduced chloride sensitivity, thus highlighting its critical role. Intriguingly, the Glu^403^-Lys^118^ salt bridge is disrupted in the complex structure of ACE bound to the inhibitory peptide BPPb (36), where Glu^403^ was seen to make a salt bridge with the P2 residue (Lys) of the peptide (Fig. 9). Thus, Glu^403^ certainly plays a role in substrate binding through the S2 subsite. The E403R mutation resulted in a loss of the strong interaction with Lys^118^, although the residues are within hydrogen bonding distance (Fig. 3*A*). Lys^118^ proved less stable (as indicated by the weak electron density for its side chain; Fig. 2*A*). Thus, E403R and the N-domain lack this stabilizing salt bridge and show lower *K_d_*_(app)_ values for chloride binding (Table 7), suggesting that the chloride pocket is in a conformation that can more readily coordinate chloride.

Further evidence of the stabilizing role of the Glu^403^-Lys^118^ interaction is seen in the change in thermodynamic parameters for E403R relative to the C-domain (Fig. 8*C*). The ΔΔ*G* values are reduced for all substrates relative to the C-domain, which is due to the observed increase in *k*_cat_/*K_m_* at 0 mm NaCl and decrease at 20 mm NaCl (Fig. 8, *A* and *B*). That the pattern and magnitude of the enthalpies (ΔΔ*H*) for the substrates correlates well with the C-domain indicates that there should be no major structural rearrangement. The lack of significant enthalpy changes do not discount minor structural differences, which are evidenced by less favorable changes in entropy (−*T*ΔΔ*S*). These minor structural effects would help explain the *K_m_* and *k*_cat_ data for E403R relative to C-domain (Table 4). E403R shows increased *k*_cat_ values at maximal activity for Z-FHL and AngI (929.34 and 9.08 s^−1^) relative to C-domain (282.00 and 7.38 s^−1^), which is countered by higher *K_m_* values in the presence of chloride for E403R (0.530 and 0.061 mm) over the C-domain (0.120 and 0.036 mm). These result in lower maximal *k*_cat_/*K_m_* values with both substrates for E403R but serve to confirm that some structural variation is present. The HHL substrate does not appear to interact strongly within the S2 region, and it is likely that its affinity is predominantly affected by S1′/S2′ interactions.

These results provide evidence for a stabilizing role for Glu^403^ in the C-domain via its interaction with Lys^118^ within the hinge-bending region rather than a functional ion-gated channel. The salt bridge serves to reduce the affinity of chloride within the chloride 2 pocket of C-domain as well as affect the structural architecture of the S2 pocket. Substrate interactions within this pocket, exemplified by Z-FHL and to a greater extent AngI, appear to disrupt the Glu^403^-Lys^118^ interaction and allow for improved chloride binding. The absence of interaction in the N-domain means that the chloride 2 pocket would have a higher affinity for chloride, even in the absence of any substrate interaction within the S2 pocket. The size of the substrate and, more importantly, how far it extends into the non-prime subsites has been shown to be important where substrate interactions in the S2 pocket modulate chloride affinity directly in the C-domain of ACE. These interactions are likely to be propagated via structural perturbations around the active site rather than any specific interaction, which is consistent with the high degree of variability in ACE substrate identity.

##### Substrate-mediated Chloride Dependence

The interactions and mechanisms within the two chloride pockets have been characterized using structural, mutagenic, and kinetic data to highlight the close relationship between the chloride dependence and substrate composition. Arg^522^ interacts indirectly with Glu^411^ and Tyr^523^, thus affecting the catalytic site by stabilizing the enzyme-substrate transition state intermediate. Importantly, the affinity of chloride coordinating Arg^522^ is moderated by structural constraint via the Glu^403^-Lys^118^ salt bridge in the C-domain of ACE, which is a point of major difference with the N-domain. Arg^522^ is part of α23, which includes other important residues for recognition of the substrate C-terminal carboxylate along with Lys^511^ situated on the 3_10_ H6 helix (36). An adjacent variable loop completes the S1 subsite and includes His^513^, probably involved in stabilizing the transition state (40); thus, minor structural movement in this area can have a large effect on catalysis and chloride binding and is influenced by substrate interactions.

**FIGURE 10.**
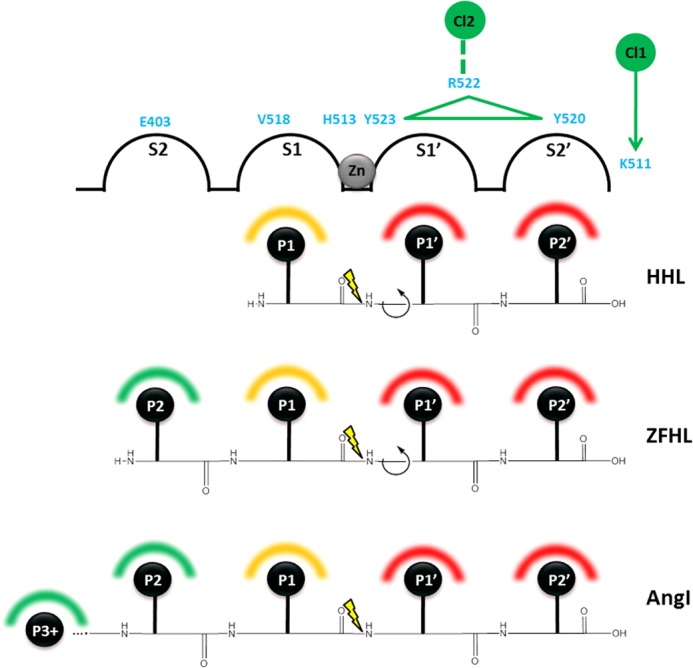
**Schematic representation of substrate binding modes in C-domain ACE.** Binding of substrate to the active site is shown using an adapted Schechter and Berger diagram (42), with active site subpockets (*S2*, *S1*, *S1*′, and *S2*′) shown as linked *half-spheres* and peptides shown as *labeled black spheres* (*P2*, *P1*, *P1*′, and *P2*′, with *P3*+ indicating residues P3–P10 for AngI). Positions of C-domain ACE residues (*light blue*) are shown *above* the relevant subsites, with helix α23 containing Arg^522^, Tyr^523^, and Tyr^520^ shown as a *green triangle*. Chloride ions (*numbered green spheres*) are shown, with chloride coordination to Arg^522^ indicated by a *dashed green line* and the influence of chloride on Lys^511^ shown by a *green arrow*. The active site zinc (*gray sphere*) indicates the position of the catalytic mechanism between S1 and S1′, with *yellow lightning* showing the position of the scissile bond for each peptide. Interactions that increase chloride affinity (*green-shaded semicircles*), those that do not increase chloride affinity (*red-shaded semicircles*), and interactions that may affect chloride affinity (*orange-shaded semicircles*) are indicated *above* each peptide residue.

The effect of substrate composition on activity can be inferred from the kinetic and structural data. The tripeptide HHL shows a highly chloride-dependent profile exemplified by a high *K_d_*_(app)_. The P1′ and P2′ residue interactions within their respective pockets have an unfavorable influence on chloride affinity, presumably via interaction or non-interaction with Tyr^523^ and Tyr^520^ (Fig. 10). Longer peptides (Z-FHL and AngI), although sharing the same P′ composition as HHL, have been shown not to reduce chloride binding. The results with both Z-FHL and AngI show a much tighter *K_d_*_(app)_ for chloride binding; thus, they are likely to moderate chloride affinity via interaction with the S2 pocket. This may be mediated by disruption of the Glu^403^-Lys^118^ salt bridge. It is possible that the greater length of AngI has a significant structural effect on the chloride pocket, which may also account for the relatively lower *k*_cat_ values.

The role of the chloride 1 pocket in C-domain ACE is less clear; however, we show that it does have some effect on chloride dependence. The chloride within this pocket may be present in a structural role, and there may not be any significant dynamic movement in this pocket. Alternatively, Moiseeva *et al.* (41) suggested that chloride binding in this pocket may have an inhibitory role at high chloride concentrations. The latter possibility agrees with our proposal, where binding of chloride could affect C-terminal carboxylate coordination within the S2′ pocket and thereby moderate activity via those interactions; however, this requires further investigation.

## CONCLUSION

Key amino acid mutations in the C-domain of ACE showed marked effects on chloride dependence when evaluated using kinetic and thermodynamic data determined for the hydrolysis of HHL, Z-FHL, and AngI under various chloride concentrations. The interpretation was complemented with the molecular details provided by the determination of the crystal structures for four of the mutants. A model of interactions in the chloride 1 pocket was developed to explain how chloride binding might modulate Lys^511^ coordination and potentially S2′ pocket conformation in the C-domain. Interactions between Arg^522^ and the zinc-coordinating Glu^411^ as well as with the transition state-stabilizing residue Tyr^523^ are described, the relevance of which was supported by interpretation of the kinetic and thermodynamic effects of the R522Q and R522K mutants. This also suggested that chloride binding was most likely affected by subtle structural effects that could be modulated by differing substrate interactions. Interpretation of the E403R mutant kinetics and thermodynamics allowed refinement of this concept and demonstrated that Glu^403^ in the C-domain of ACE plays a role in reducing chloride affinity in the chloride 2 pocket via a salt bridge with Lys^118^, an interaction that is not present in the N-domain and which probably represents the primary differentiation in chloride dependence between the domains. Finally, the different ways in which substrate interactions can modulate chloride affinity via the S′ or S2 pockets are described. This study has provided a framework to describe chloride dependence of the ACE N- and C-catalytic sites and will contribute considerably to substrate selectivity studies as well as the development of domain-selective inhibitors for the improved treatment of hypertension (C-selective ACEi) and fibrosis (N-selective ACEi).

## Footnotes

5 The abbreviations used are:

ACE: angiotensin-I-converting enzyme
sACE: somatic angiotensin-converting enzyme
tACE: testicular ACE
AngI and AngII: angiotensin I and II, respectively
ITC: isothermal titration calorimetry
HHL: hippuryl-l-His-Leu
Z: benzyloxycarbonyl
SA: specific activity
BppB: bradykinin potentiating peptide B
TAPSO: 3-[[1,3-dihydroxy-2-(hydroxymethyl)propan-2-yl]amino]-2-hydroxypropane-1-sulfonic acid.
